# Health policy and planning-thank you to reviewers

**DOI:** 10.1093/heapol/czag031

**Published:** 2026-04-17

In 2025, *Health Policy and Planning* received over 950 submissions from over 110 countries. It published more than 100 papers across 10 issues, providing important research, reviews, and debates, on topics relevant to health policies in low- and middle-income countries. *Health Policy and Planning* relies heavily on reviewers to provide expert and high-quality reviews. They help ensure the accuracy, quality, and credibility of the research the journal publishes. The Editors in Chief and Section Editors would like to express gratitude and appreciation for the reviewers time and effort in providing this invaluable service and continuing to do so despite high workloads. *Health Policy and Planning* full list of reviewers from 2025 is published below. We hope you continue to guide and support health policy and health system development in low- and middle-income countries in this way, and we value your support each and every year.

Chakraborty Sarbani

Syed Shahid Abbas

Dawit Abebe

Gilbert Abotisem Abiiro

Seye Abimbola

Patience Abor

Alayne Adams

Alex Adjagba

Koki Agarwal

Prince Agwu

Brandon Ah Tong

Ahmad Ahmadi Teymourlouy

Syed Ahmed

Jill Åhs

Praveenkumar Aivalli

Baktygul Akkazieva

Daniel Ali

Olakunle Alonge

Sergi Alonso

Otuto Amarauche Chukwo

Ana Amaya

Woldekidan Amde

Samuel Ankomah

Laura Anselmi

Carl Abelardo Antonio

Marco Antonio

Helen Anyasi

Edson Araujo

Gwen Arnold

Sophie Arseneault

Abdul Kalam Azad

Max Bachmann

David Baguma

Florence Baingana

Priya Balasubramaniam

Sarah Bales

Amanda Banda

Alka Barua

Ivyrose Baysic

Allison Beattie

Tekabe Belay

Stephen Bell

Zakaria Belrhiti

David Berlan

Himani Bhakuni

Barri Blauvelt

Robert Borst

Krishna Bose

Jorn Braa

Hazel Bradley

Erica Breuer

Chloe Brooks

Helen Burchett

Nicola Burger

Kent Buse

Chang Cai

Sebastiano Caleffi

Fiona Campbell

Aisha Casoojee

Alejandro Cerón

Cyndirela Chadambuka

Peipei Chai

Arpita Chakraborty

Kalipso Chalkidou

Olivia Chan

Li Chang

Collins Chansa

Eliana Chavarria Pino

Yu Chen

Qinglu Cheng

Terence Cheng

Matthew Chersich

Vesper Chisumpa

Shakira Choonara

Hong-Ling Chu

Svea Closser

Olive Cocoman

Joseph Collins

Christopher Colvin

Alison Comfort

David Contreras Loya

Sarah Cook

Ana Correa-Ossa

Joanne Csete

Sara Dada

Elina Dale

Sarah Dalglish

K. Damrongplasit

Karen Daniels

Teguh Dartanto

Mike Daube

Sara Davies

Shelby Davies

Thomas De Hoop

Karen Devries

Erica Di Ruggiero

Hassan Dib

Fahdi Dkhimi

Surabhi Dogra

Olena Doroshenko

Gifty Dufie Ampofo

Eliudi Eliakimu

Lana Elliott

Isabella Epiu

Jing Fang

Brooke Farrenkopf

Bola Fatusin

Fato Fene

Xing Lin Feng

Gunther Fink

Steffen Flessa

Ashley Fox

Jed Friedman

Hongqiao Fu

Jacopo Gabani

Dominic Dormenyo Gadeka

Thomas Gadsden

Bernhard Gaede

Rakhal Gaitonde

Jianguo Gao

Juan David García Corchero

Meenakshi Gautham

Nilesh Gawde

Jinsong Geng

Asha George

Philipos Gile

Brynne Gilmore

Ayanda Gina

Douglas Glandon

Srinivas Goli

Judite Gonçalves

Shiwei Gong

Miguel Gonzalez-Block

Wendy Gonzalez

George Gotsadze

Andrew Gray

Nicola Gray

Scott Greer

Karen Grepin

Stefanny Guerra

Panchali Guha

Lorna Guinness

Bingqing Guo

Prateek Gupta

Manon Haemmerli

Brittany Hagedorn

Jeannie L. Haggerty

Su Myat Han

Xinxin Han

Farhana Haque

Ping He

Olivia Heller

Mark Hellowell

Anna Helova

Olivia Herlinda

Alex Hill

David Hipgrave

Jens Holst

Ayako Honda

David Imbago

Collins Iwuji

Martins Iyekepolor

Adit Iyer

Debra Jackson

Nisha Jacob

Humayun Javed

Charity Jensen

Junnan Jiang

Wei Jiang

Limei Jing

Taufique Joarder

William Joe

Lakshmi Joysula

Nancy Kagwanja

Anuska Kalita

Vikash Keshri

Mishal Khan

Mary Kinney

Baridalyne Knongkynrih

James Knowles

Augustina Koduah

Antony Kollannur

Adam Koon

George Kosimbei

Catarina Krug

Emmanuel Kumah

Dinesh Kumar

Michael Kunnuji

Duncan Kwaitana

Xiaozhen Lai

Ganesh Lakshmanan

Dorothy Lall

Joni Lariat

Yong Yi Lee

Uta Lehmann-Grube

Rolando Leiva

Raphael Lencucha

Anthea Lesch

Elizabeth Lightfoot

Shea Littlepage

Mengyun Liu

Shimeng Liu

Siyuan Liu

Xinyi Liu

Marco Liverani

Harvy Joy Liwanag

James Macinko

Ajay Mahal

Marshall Makate

Segolene Malengreaux

Yexin Mao

Tiara Marthias

Celia Matyanga

Carl May

Leigh McClarty

David McCoy

Gerry McGivern

Avery McNair

Marie Meudec

Pierre Miege

Masoud Mirzaei

Shinjini Mondal

Doreen Montag

Herryman Moono

Rosemary Morgan

Alec Morton

Cheryl Moyer

Dirk Mueller

Oscar Mujica

Mulenga Mukanu

Fidele Mukinda

F. Mukumbang

Moses Mukuru

Heather Munthe-Kaas

Kyaw Myat Thu

Nellie Myburg

Rose Nanyonga

Tom Newton-Lewis

Claire Nightingale

Jing Ning

Muhammad Naveed Noor

Andrea Nove

Emmanuel Nshakira-Rukundo

Owen Nyamwanza

Thomas O’Connell

Francis Obare

Emmanuel Odame

Tomson Ogwang

Ida Okeyo

Adeyemi Okunogbe

Gladys Olisaekee

Dorothy Oluoch

Marsha Orgill

Emanuel Orozco

Evans Otieku

Neelaveni Padayachee

Ligia Paina

Bhuputra Panda

Sarita Panday

Justin Parkhurst

Ke Peng

Janet Perkins

John Porter

James Potter

Madan Mohan Pradhan

Phusit Prakonsai

Anni-Maria Pulkki-Brännström

Victor Quesada-Cubo

Amanda V. Quintana

A. Venkat Raman

Arkalgud Ramaprasad

Cesar Renteria

Danielle Resnick

Brian Rice

Diego Ríos-Zertuche

Lesley Robertson

Sarah Rozenblum

Simon Rushton

Belén Saenz-de-Miera

Ramiro Salas

Dell Saulnier

Joanna Schellenberg

Helen Schneider

Katherine Semrau

Donat Shamba

Jayendra Sharma

Parth Sharma

Jeremy Shiffman

Leah Shipton

Surbhi Shrivastava

Zubin Shroff

Maylene Shung-King

Yafei Si

Pooja Singh

Shalini Singh

Jackline Sitienei

Julia Smith

Stephanie Smith

Emily South

Susan Sparkes

Paula Spinola

Veena Sriram

Swati Srivastava

Elizabeth Stierman

Preslava Stoeva

Daniel Strachan

Emmanuel Streel

Gang Sun

Naiema Taliep

Mei Jin Melisa Tan

Tara Tancred

Ajay Tandon

Gina Teddy

Fabrizio Tediosi

Marieke Theron

Collins Timire

Stephanie Topp

Michael Touchton

Jean-François Trani

Emily Treleaven

Judyth L. Twigg

Beena Varghese

Eduardo Viegas da Silva

Stephen Vosti

Marius Wamsiedel

Hong Wang

Xiaohui Wang

David Watkins

Evelyn Waweru

Jinglin Wen

Woranan Witthayapipopsakul

Dadong Wu

Jing Wu

Biao Xu

Jin Xu

Qiang Yao

Peter Badimak Yaro

Vijayshree Yellappa

Lufa Zhang

Qiong Zhang

Yuxi Zhang

Menghan Zhao

Xiaoying Zhu

Petmore Zibako

